# Comparative Toxicity of Antifouling Paint Particle Suspensions and Dissolved Leachates in the Marine Copepod *Tigriopus japonicus*

**DOI:** 10.3390/toxics14100890

**Published:** 2026-10-06

**Authors:** Sung-Eun Hong, Bhavya Kachiprath, Duck-Hyun Kim, Harim Jeong, Chae Hwa Kim, Tae Hee Kim, Chang-Bum Jeong

**Affiliations:** 1 Department of Marine Science, College of Natural Sciences, Incheon National University, Incheon 22012, Republic of Korea; 7940hse@inu.ac.kr (S.-E.H.); bhavyanirmalagiri@gmail.com (B.K.); dhkim5@inu.ac.kr (D.-H.K.); harimxx@inu.ac.kr (H.J.); 2 Textile Innovation R&D Department, Korea Institute of Industrial Technology, Ansan 15588, Republic of Korea; purech@kitech.re.kr (C.H.K.); thkim75@kitech.re.kr (T.H.K.)

**Keywords:** antifouling paint particles, booster biocides, leachate, copepod, transcriptome

## Abstract

Antifouling paint particles (APPs), generated by coating degradation and ship-maintenance activities, are an emerging but poorly characterized source of marine pollution. APPs can release particle-associated and dissolved toxicants, yet their relative contributions to toxicity remain unclear. In this study, environmentally collected APPs were used to prepare two exposure media: an APP suspension containing suspended paint particles and dissolved constituents, and a corresponding membrane-filtered leachate representing the operationally defined dissolved fraction. Their toxic effects on the marine copepod *Tigriopus japonicus* were assessed using acute toxicity tests, chronic life-history assays, and comparative transcriptomic analyses. The APP suspension caused greater acute and chronic toxicity than the leachate under the test conditions. Transcriptomic profiling revealed 474 and 116 differentially expressed genes (DEGs) following APP suspension and leachate exposure, respectively, compared with the control. Gene Ontology (GO) enrichment and toxicity pathway analyses identified both shared and exposure-specific responses, with broader perturbations of toxicity-related biological processes following APP suspension exposure. Collectively, the greater phenotypic responses and distinct molecular profiles highlight the importance of considering multiple exposure forms when evaluating APP toxicity.

## 1. Introduction

Marine biofouling poses a persistent challenge to maritime operations by increasing hydrodynamic drag, fuel consumption, greenhouse gas emissions, and maintenance costs. Antifouling coatings are therefore routinely applied to ship hulls to prevent the settlement and growth of fouling organisms. Following the global prohibition of tributyltin (TBT)-based coatings, modern antifouling paints have evolved into chemically complex formulations that typically combine copper-based active ingredients with booster biocides, such as zinc pyrithione and copper pyrithione, as well as other proprietary additives [1,2,3]. Although these formulations have reduced the environmental risks associated with organotin compounds, the continuous release of biocides and other coating constituents remains an important source of contamination in marine ecosystems.

Antifouling paint-derived contaminants enter aquatic environments through continuous leaching during vessel operation, as well as through the physical release of coating materials caused by surface weathering, hull cleaning, dry-docking, and high-pressure water-jet blasting during ship maintenance [4,5,6,7]. These processes release both dissolved constituents and antifouling paint particles (APPs), creating a complex exposure scenario in which marine organisms may encounter dissolved toxicants, suspended particles, and particle-associated contaminants simultaneously. Unlike dissolved compounds, APPs may remain suspended in the water column or accumulate in sediments, where they can continue to release associated chemicals while also serving as particulate vectors that may be ingested by aquatic organisms [8,9]. Commercial antifouling formulations are generally proprietary and chemically complex, comprising polymeric binders, pigments, metal-based additives, and organic or organometallic booster biocides [10,11,12]. Accordingly, environmentally released APPs should be regarded as complex contaminant systems rather than as simple sources of individual chemicals, with their toxicity potentially arising from the combined effects of the particles themselves, particle-associated contaminants, and constituents released into the surrounding water. Nevertheless, the biological responses to particle-containing APP suspensions and their corresponding membrane-filtered leachates remain incompletely characterized. Direct comparisons between particle-containing APP suspensions and their corresponding dissolved fractions are therefore needed to determine whether assessments based solely on leachates underestimate the ecological hazards posed by APPs. 

The marine harpacticoid copepod *Tigriopus japonicus* is widely used as a model organism in marine ecotoxicology because of its ecological relevance, short generation time, ease of laboratory culture, and sensitivity to a broad range of environmental contaminants [13,14,15]. As a common member of coastal meiofaunal communities, *T. japonicus* occupies an important trophic position linking primary producers and detrital resources to higher-level consumers, making it an ecologically relevant indicator of contaminant-induced effects in coastal ecosystems. Its distinct postembryonic developmental stages, sexual dimorphism, high fecundity, and ability to produce multiple sequential broods following a single mating event also make it particularly suitable for evaluating chronic life-history effects [13,16,17]. Furthermore, the availability of a fully annotated reference genome and established transcriptomic resources facilitates the integration of organism-level toxicity endpoints with molecular response profiles, thereby providing mechanistic insights into contaminant-induced biological perturbations [18,19,20].

In the present study, environmentally collected APPs were used to prepare two matched exposure media: (i) an APP suspension containing suspended paint particles and dissolved constituents and (ii) the corresponding membrane-filtered APP leachate, representing the dissolved fraction. Acute and chronic in vivo assessments were conducted to characterize organism-level effects, while comparative transcriptomic analyses were performed to identify the molecular response patterns associated with each exposure. By directly comparing the two exposure media, we sought to determine whether the presence of suspended APPs increased toxicity beyond the effects caused by dissolved constituents alone. Through the integration of phenotypic and transcriptomic endpoints, this study provides a more comprehensive understanding of the biological and molecular responses to environmentally released APPs and emphasizes the need to incorporate both particulate and dissolved exposure pathways into marine ecological risk assessments.

## 2. Materials and Methods

### 2.1. Collection and Processing of Antifouling Paint Particles

Antifouling paint particles (APPs) were collected during water-jet blasting of the hull of the research vessel *R/V ISABU*, operated by the Korea Institute of Ocean Science and Technology (KIOST). The blasting residues were collected without prefiltration, after which the settled bottom fraction was recovered by natural density separation and transferred to the Korea Institute of Industrial Technology (KITECH) for further processing. The recovered material was frozen at −20 °C for 24 h and subsequently vacuum-dried at 30 °C for 72 h. After drying, the resulting APP powder was collected and gently mixed to improve sample homogeneity prior to further analysis. 

For particle-size analysis, the dried APP powder was dispersed in distilled water and subsequently vortexed and ultrasonicated to facilitate particle dispersion. Particle-size distributions were determined using a laser diffraction particle-size analyzer (LS 13320; Beckman Coulter Inc., Brea, CA, USA). The morphology of the APPs was examined using field-emission scanning electron microscopy (FE-SEM; SU8000, Hitachi, Tokyo, Japan). Images were acquired at an accelerating voltage of 5.0 kV, a working distance of 7.9 mm, and a magnification of ×300. The composition and physicochemical properties of the source antifouling paint were obtained from the available Material Safety Data Sheet (MSDS) and Technical Data Sheet (TDS). The reported product information, together with available toxicity threshold data for the listed constituents, is summarized in Table S1.

### 2.2. Preparation of APP Exposure Media

**Figure 1:**
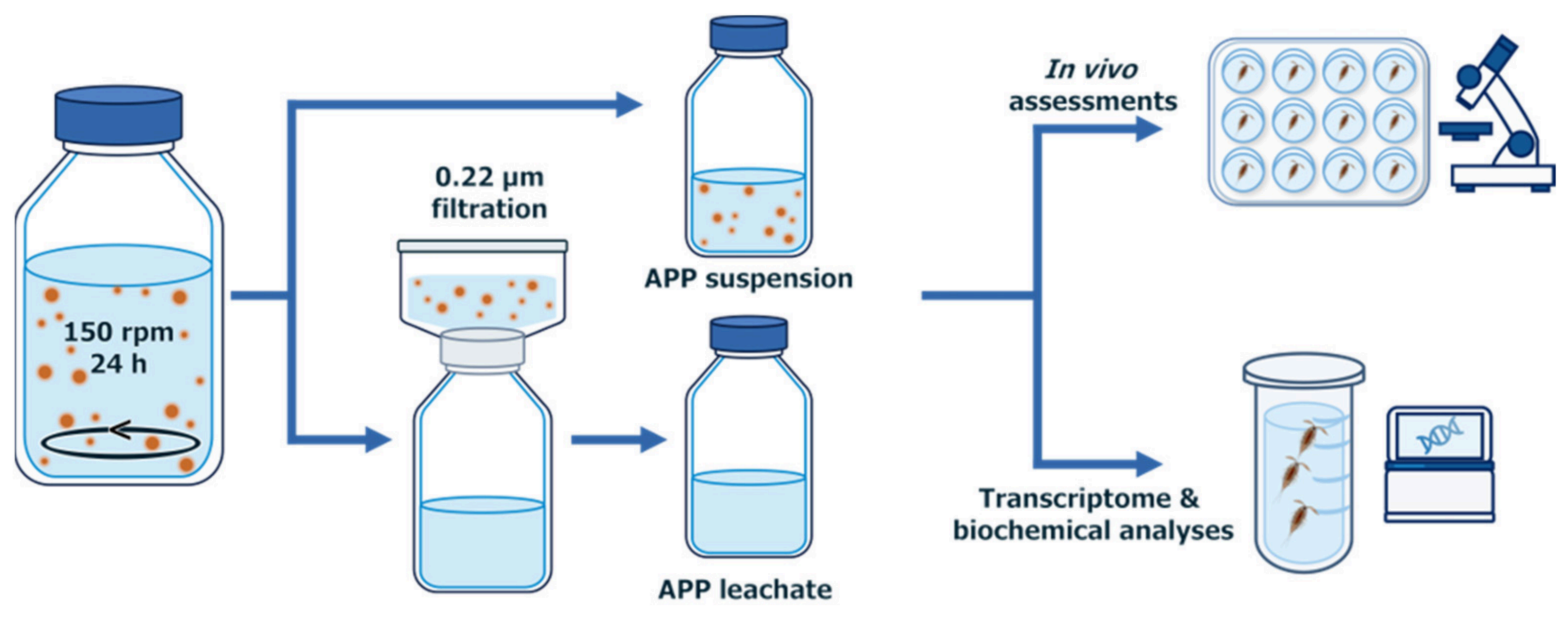
Schematic representation of the experimental workflow. APPs were suspended in artificial seawater and stirred at 150 rpm for 24 h. The resulting APP suspension was used directly or filtered through a 0.22-μm membrane to obtain the corresponding leachate for in vivo toxicity tests and transcriptomic analyses.

The parent APP medium was prepared by suspending 25 mg of APPs in 500 mL of artificial seawater (ASW; 30 psu), corresponding to an initial nominal loading of 50 mg L^−1^. The suspension was continuously stirred at 150 rpm for 24 h using a magnetic stirrer to promote the release of leachable constituents from the particles (Figure 1). Test concentrations were prepared by dilution of this parent medium and are therefore reported as nominal APP concentrations based on the initially added APP mass. After the 24-h leaching period, the suspension was divided into two equal aliquots. One 250 mL aliquot was retained without filtration as the APP suspension, containing both suspended paint particles and dissolved constituents. The remaining 250 mL aliquot was vacuum-filtered through a 0.22-μm MF-Millipore™ membrane filter (Merck, Darmstadt, Germany), and the resulting filtrate was designated the APP leachate, representing the dissolved fraction. Both exposure media were transferred to pre-sterilized amber screw-cap vials and stored until use.

### 2.3. In Vivo Toxicity Assessment

The marine harpacticoid copepod *T. japonicus* used in this study was originally collected from Uljin, South Korea (36°58′43.01″ N, 129°24′28.40″ E). Species identity was confirmed based on morphological characteristics and mitochondrial cytochrome *c* oxidase subunit I (*COI*) gene sequencing [21]. Laboratory cultures were maintained in artificial seawater (ASW; 30 psu) filtered through a 0.22-µm membrane filter (Corning Co., New York, NY, USA) at 25 °C under a 12:12-h light:dark photoperiod. Copepods were fed daily with the green microalga *Tetraselmis* sp. at 6.0 × 10^4^ cells mL^−1^. The population was maintained under these conditions for several generations before use in the experiments. Prior to APP exposure, copepods were acclimated to the experimental conditions for 4 h.

The acute toxicity of the APP suspension and leachate was evaluated separately using adult male *T. japonicus*. Ten randomly selected individuals were transferred to each well of a 24-well culture plate containing 4 mL of the corresponding test medium. Nominal exposure concentrations were 0, 5, 10, 15, and 20 mg APP L^−1^ for the APP suspension and 0, 10, 20, 30, 40, and 50 mg APP-equivalent L^−1^ for the leachate. Leachate concentrations were expressed on an APP-equivalent basis according to the mass of APPs used to prepare the parent leachate. The 0 mg L^−1^ treatment contained only ASW and served as the control. No food was provided during the exposure period, in accordance with established acute toxicity testing procedures for *T. japonicus* [22]. Mortality was recorded after 24 and 48 h using a stereomicroscope (SZX7; Olympus, Tokyo, Japan). Median lethal concentrations (LC_50_) and concentrations causing 10% mortality (LC_10_) were estimated by probit analysis using ToxRat software (version 2.09; ToxRat Solutions GmbH, Alsdorf, Germany).

Chronic toxicity tests were conducted using newly hatched *T. japonicus* nauplii (<6 h post-hatching). Nauplii were exposed to APP suspension concentrations of 0.1068, 0.534, and 1.068 mg APP L^−1^ or APP leachate concentrations of 0.7416, 3.7082, and 7.4165 mg APP-equivalent L^−1^. These concentrations corresponded to 1/20, 1/4, and 1/2 of the respective LC_10_ values derived from the acute toxicity tests. At the beginning of each experiment, ten nauplii were randomly transferred to each well of a 24-well culture plate containing 4 mL of the corresponding test medium. Control organisms were maintained under identical conditions in 0.22-µm-filtered ASW without APP exposure. 

Developmental progression was monitored daily using a stereomicroscope during water renewal, with additional observations at approximately 3–24 h intervals around the nauplius-to-copepodid and copepodid-to-adult transitions. Developmental time was recorded in hours from exposure initiation to the observed transition and expressed as the time required for nauplius-to-copepodid (N–C) and nauplius-to-adult (N–A) development. Some mortality occurred during the initial nauplius setup, irrespective of treatment or control, and these individuals were excluded from developmental-time calculations. No mortality was observed after nauplius stage II. Developmental responses were analyzed using the replicate wells as the experimental units. During the chronic exposure, copepods in both control and APP-exposure media were fed daily with *Tetraselmis* sp. at 6.0 × 10^4^ cells mL^−1^, and approximately 50% of the test medium was renewed daily to maintain exposure conditions. The algae were not pre-exposed to APPs or leachates before being added to the test media. All toxicity tests were conducted with three replicate wells per treatment, each containing ten individuals.

### 2.4. RNA Extraction, cDNA Library Construction and Sequencing 

For transcriptomic analysis, approximately 200 adult *T. japonicus* were exposed for 24 h to 50 mL of APP suspension (1.068 mg APP L^−1^) and APP leachate (7.4165 mg APP-equivalent L^−1^). These concentrations corresponded to the highest sublethal concentrations used in the chronic toxicity assays. Three replicate exposure groups were prepared for each treatment, including the control. Following exposure, total RNA was extracted using TRIzol reagent (Invitrogen, Carlsbad, CA, USA) according to the manufacturer’s instructions and stored at −80 °C until further analysis. RNA concentration and purity were assessed using a QIAxpert spectrophotometer (QIAGEN, Hilden, Germany), and RNA integrity was evaluated using an Agilent 2100 Bioanalyzer (Agilent Technologies, Waldbronn, Germany). Only RNA samples with an RNA integrity number (RIN) greater than 7 were used for library preparation.

For library preparation, messenger RNA (mRNA) was enriched from 1 µg of total RNA using oligo(dT)-conjugated magnetic beads and subsequently fragmented. The fragmented mRNA was reverse-transcribed into complementary DNA (cDNA) using random hexamer primers. Strand-specific cDNA libraries were generated by polymerase chain reaction (PCR) amplification, and library quality was assessed by capillary electrophoresis using an Agilent Bioanalyzer (Agilent Technologies, Inc., Santa Clara, CA, USA). Library concentrations were determined by quantitative PCR using SYBR Green PCR Master Mix (Applied Biosystems, Foster City, CA, USA). Indexed libraries were pooled at equimolar concentrations, and clusters were generated on an Illumina cBot system. The pooled libraries were subsequently sequenced on an Illumina NovaSeq 6000 platform to generate 100-bp paired-end reads.

### 2.5. Bioinformatic Analysis

Raw sequencing reads were quality-filtered and trimmed before being mapped to the *T. japonicus* reference genome [19]. Gene-level read counts were generated using htseq-count implemented in HTSeq (version 1.0) [23]. Differential gene expression analysis was performed using the edgeR package in R [24], with library sizes normalized using the trimmed mean of M-values (TMM) method [25]. Differentially expressed genes (DEGs) were defined as those exhibiting an absolute fold change of at least 1.5 and a false discovery rate (FDR)-adjusted *p* value < 0.1. Functional annotation of DEGs was performed using BLASTX searches against the UniProtKB/Swiss-Prot database (release 2024_06), with an E-value cutoff of 1 × 10^−5^. Gene Ontology (GO) enrichment analysis for biological process terms was conducted using DAVID, and semantically redundant GO terms were summarized using REVIGO [26,27]. Enrichment significance was evaluated based on the raw enrichment *p* values reported by DAVID, with *p* < 0.05 considered statistically significant. All annotated DEGs were subsequently analyzed using Ingenuity Pathway Analysis (IPA; QIAGEN, Hilden, Germany) to identify toxicity-related pathways and gene interaction networks. Complete lists of DEGs, enriched GO terms, and IPA toxicity-related pathways are provided in Tables S2–S4.

### 2.6. Statistical Analysis 

All data are presented as the mean ± standard deviation (SD). Prior to parametric analysis, data were assessed for normality and homogeneity of variances using the Shapiro–Wilk and Levene’s tests, respectively. Differences among treatment groups (comparisons of continuous biological endpoints) were evaluated using one-way analysis of variance (ANOVA), followed by Tukey’s honestly significant difference (HSD) post hoc test for pairwise comparisons. Statistical significance was set at *p* < 0.05. All statistical analyses were performed using IBM SPSS Statistics version 27 (IBM Corp., Armonk, NY, USA).

## 3. Results

### 3.1. Particle-Size Characterization of Antifouling Paint Particles 

**Figure 2:**
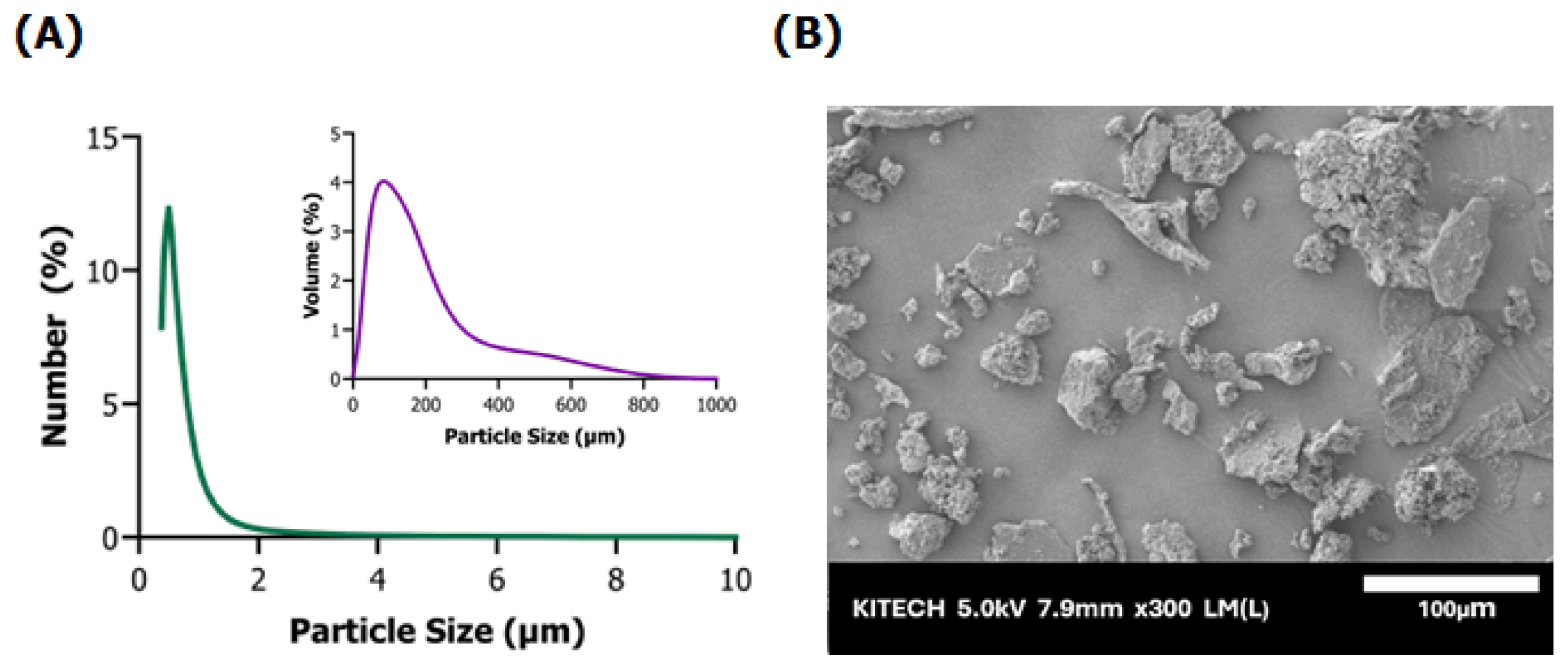
(**A**) Volume- and number-based particle-size distributions of APPs. (**B**) The morphology of APPs observed by field-emission scanning electron microscopy (FE-SEM).

Particle-size analysis revealed a volume-weighted mean diameter of 112.1 ± 94.0 µm and a number-weighted mean diameter of 0.712 ± 0.696 µm (Figure 2). Relatively large paint flakes, approximately 100 µm in diameter, accounted for most of the total particle volume, whereas submicron particles (<1 µm) dominated the particle number distribution. These contrasting distributions indicate that the APP sample comprised a highly heterogeneous particle population consisting of large paint fragments and numerous fine particles.

### 3.2. In Vivo Effects of APP Suspension and Leachate 

**Figure 3:**
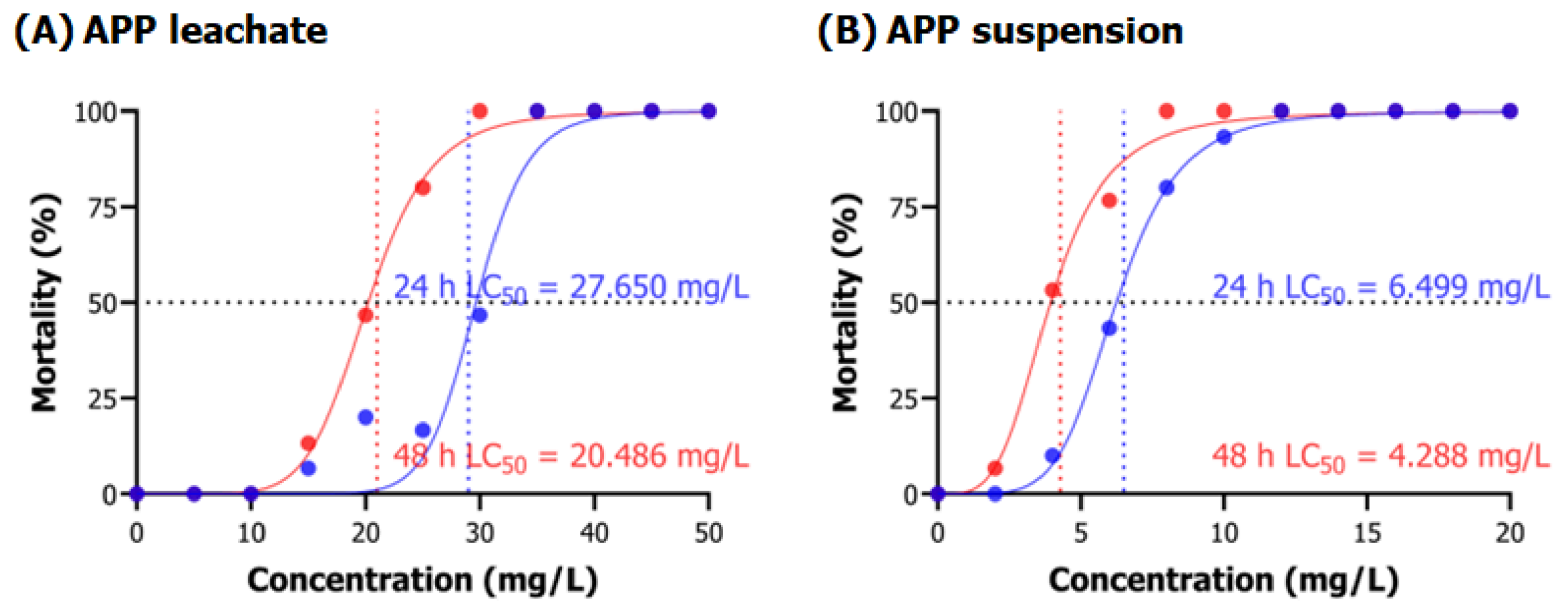
Acute toxicity of antifouling paint particles (APP) exposure media to adult *Tigriopus japonicus*. Concentration–response relationships and estimated LC_50_ values for (**A**) the APP leachate and (**B**) the APP suspension following 24 h (red dotted line) and 48 h (blue dotted line) of exposure.

The APP suspension exhibited greater acute toxicity to adult *T. japonicus* than the corresponding leachate at both exposure durations. Mortality increased in a concentration- and time-dependent manner under both exposure conditions. The APP leachate induced concentration-dependent mortality, with LC_50_ values decreasing from 27.650 mg APP-equivalent L^−1^ (95% CI: 25.090–30.383) at 24 h to 20.486 mg APP-equivalent L^−1^ (95% CI: 19.198–21.776) at 48 h (Figure 3A). In comparison, the APP suspension produced equivalent effects at substantially lower concentrations, with LC_50_ values of 6.499 APP L^−1^ (95% CI: 5.951–7.039) and 4.288 mg APP L^−1^ (95% CI: 3.780~4.790) at 24 and 48 h, respectively (Figure 3B). Accordingly, the LC_50_ values of the APP suspension were approximately 4.3- and 4.8-fold lower than those of the leachate at 24 and 48 h, respectively. Probit regression parameters, including slopes and goodness-of-fit statistics, are provided in Table S5.

**Figure 4:**
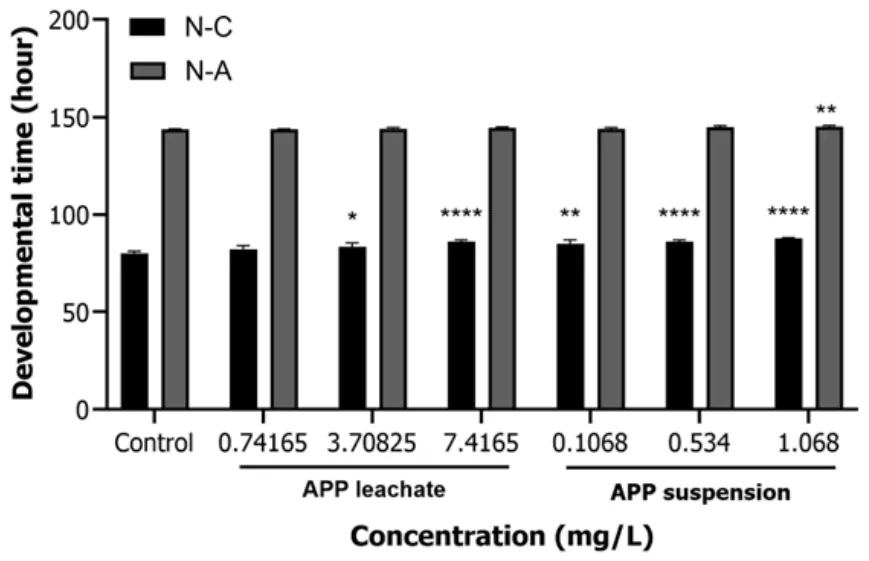
Effects of chronic exposure to APP suspension and leachate on the development of *Tigriopus japonicus*. Development time was assessed based on the nauplius-to-copepodid (N-C) and nauplius-to-adult (N-A) transitions. Data are presented as mean ± SD (*n* = 3). Asterisks indicate significant differences from the control: * *p* < 0.05, ** *p* < 0.01, and **** *p* < 0.0001.

Chronic exposure to both the APP suspension and leachate altered the developmental progression of *T. japonicus* (Figure 4). The nauplius-to-copepodid (N–C) developmental period was prolonged in a concentration-dependent manner under both exposure conditions, indicating that the early developmental transition was particularly sensitive to APP exposure. In contrast, the nauplius-to-adult (N–A) developmental period was significantly prolonged only at the highest APP suspension concentration, whereas none of the leachate treatments caused a significant delay.

### 3.3. Transcriptomic Responses to APP Suspension and Leachate 

**Figure 5:**
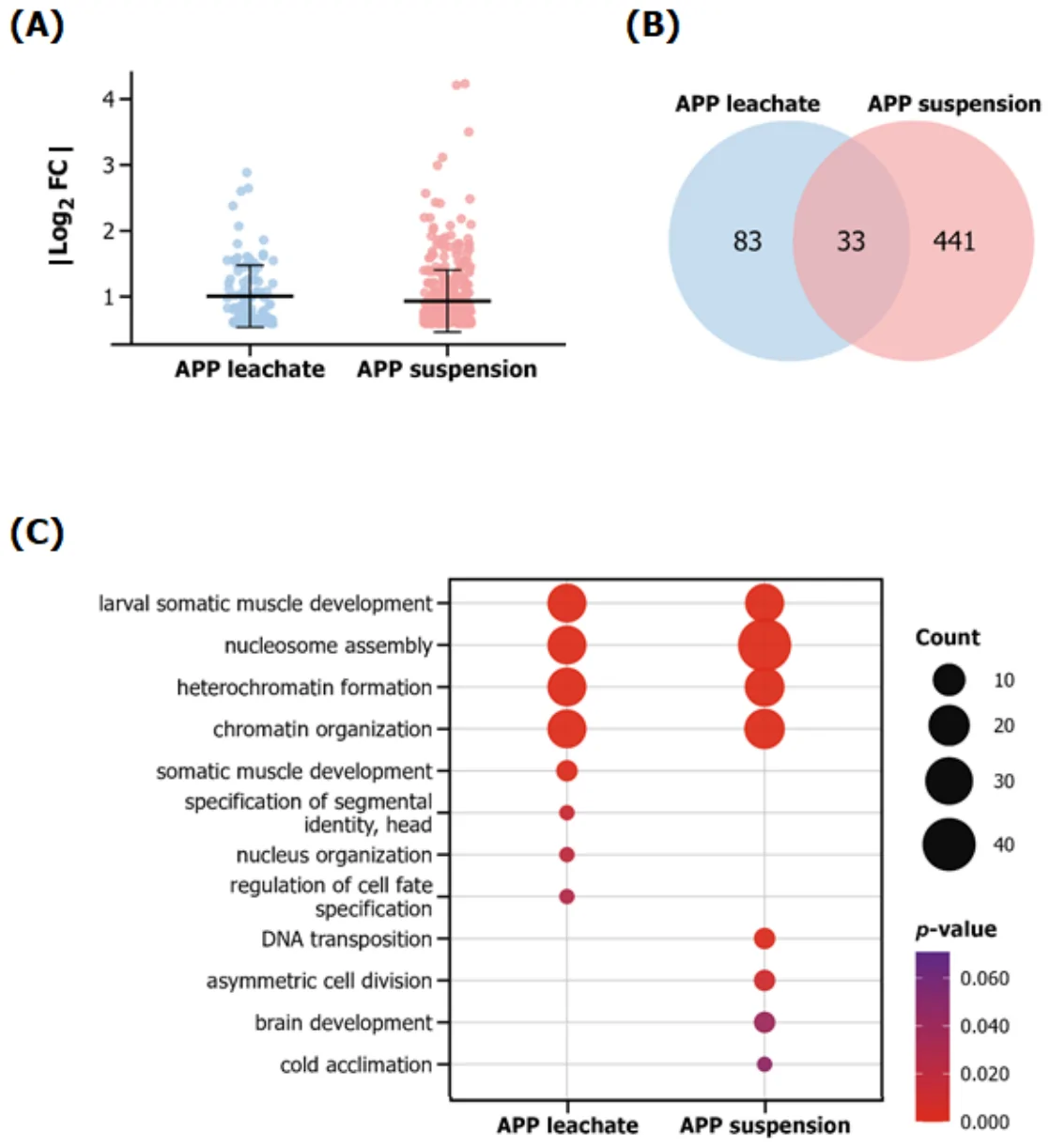
Transcriptomic responses of *Tigriopus japonicus* following 24-h sublethal exposure to APP suspension and leachate. (**A**) Distribution of absolute log_2_ fold changes (|log_2_FC|) of differentially expressed genes (DEGs) (both upregulated and downregulated genes) in the APP suspension and leachate treatments relative to the control. Each datapoint represents an individual DEG. (**B**) Venn diagram showing the overlap of DEGs identified in the APP suspension and leachate treatments. The numbers indicate treatment-specific and shared DEGs between the two exposure groups. (**C**) Gene Ontology (GO) biological process enrichment analysis of DEGs in *T. japonicus* following APP suspension and leachate exposure. The bubble plot shows enriched GO biological processes, with bubble size representing the number of DEGs associated with each GO term and bubble color indicating enrichment significance (*p*-value).

Transcriptomic profiling revealed distinct gene expression responses following 24-h exposure to the APP suspension and leachate. Compared with the control, the APP suspension induced a substantially broader transcriptional response than the leachate, as evidenced by both a greater number of differentially expressed genes (DEGs) and larger absolute log_2_ fold-change values of the DEGs identified in each comparison (Figure 5A). Compared with the control, 474 and 116 DEGs were identified following exposure to the APP suspension and leachate, respectively (Figure 5B). Among these, 33 genes met the DEG criteria in both comparisons, whereas 441 and 83 genes met the criteria only in the suspension-versus-control and leachate-versus-control comparisons, respectively.

Gene Ontology (GO) enrichment analysis identified 12 distinct biological process terms with raw *p* values < 0.05 across the APP suspension and leachate groups (Figure 5C). Eight significantly enriched GO terms were identified in each treatment group. Four terms—nucleosome assembly, larval somatic muscle development, heterochromatin formation, and chromatin organization—were shared between the two exposure groups, suggesting common effects on chromatin-related and developmental processes. In the APP suspension group, additional enriched terms included DNA transposition, asymmetric cell division, brain development, and cold acclimation. In the leachate group, additional enriched terms included somatic muscle development, specification of segmental identity in the head, nucleus organization, and regulation of cell-fate specification. Overall, the GO enrichment analysis revealed both shared and distinct biological process annotations between the APP suspension and leachate DEG datasets.

**Figure 6:**
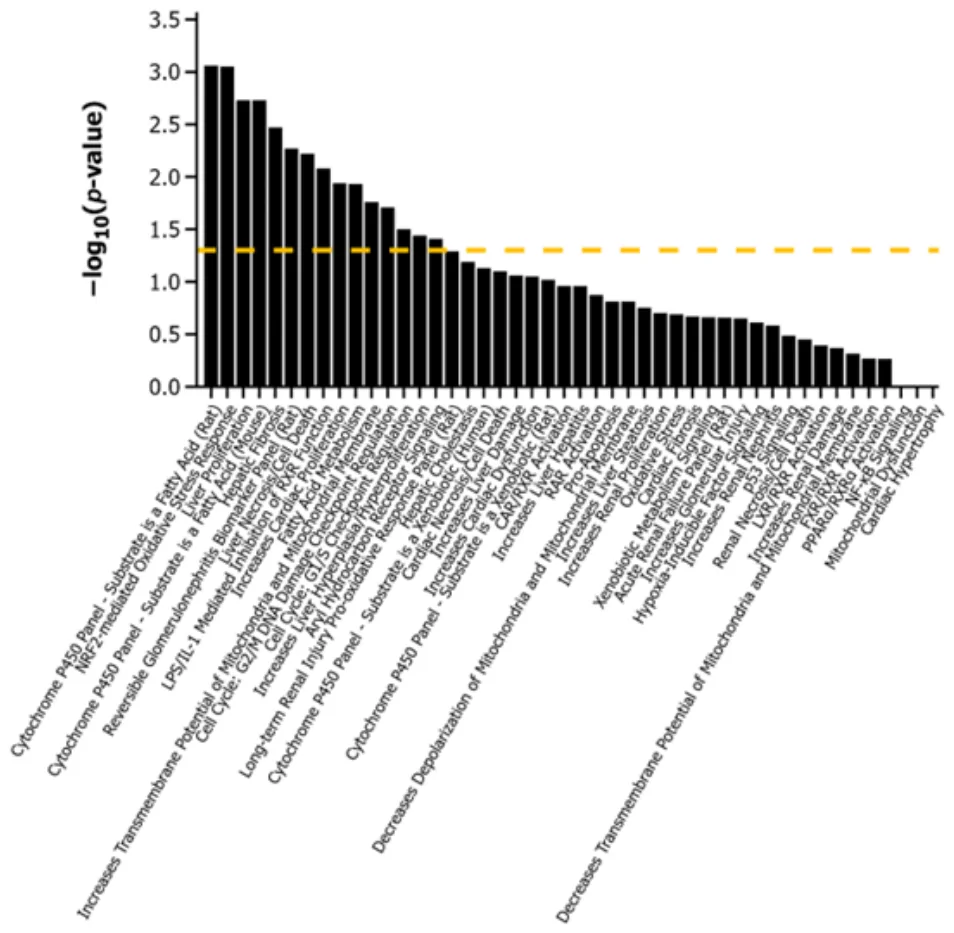
Ingenuity Pathway Analysis (IPA) Toxicity List enrichment of differentially expressed genes (DEGs) in *Tigriopus japonicus* following exposure to the APP suspension. The yellow dashed line represents the significance threshold (−log_10_(*p*-value) = 1.3; equivalent to *p* = 0.05).

Ingenuity Pathway Analysis (IPA) Tox List analysis identified significantly enriched toxicity-related categories only in the APP suspension group (Figure 6). The enriched categories were associated primarily with xenobiotic biotransformation, oxidative stress, mitochondrial function, and cell-cycle regulation. Prominent categories included cytochrome P450-mediated fatty acid metabolism and the NRF2-mediated oxidative stress response. Additional enriched categories were related to cell-cycle DNA damage checkpoint regulation and mitochondrial membrane function. Collectively, these enrichment patterns indicate that APP suspension exposure was associated with molecular perturbations involving detoxification, redox homeostasis, mitochondrial function, and cellular stress responses.

## 4. Discussion

The APP suspension caused substantially greater organism-level toxicity and transcriptional perturbation in *T. japonicus* than the corresponding leachate under the tested exposure conditions. Although both exposure media were prepared from the same environmentally collected APP material, the particle-containing suspension consistently induced greater acute mortality and more pronounced developmental impairment than the membrane-filtered leachate, indicating that the two exposure preparations produced distinct biological responses. However, because the chemical composition of the exposure media was not quantitatively characterized, the greater responses observed with the suspension cannot be attributed specifically to the physical presence of particles. Particle-size characterization further revealed a highly heterogeneous APP population comprising relatively large paint flakes and numerous submicron particles. Owing to their high surface-area-to-volume ratios and mobility, these fine particles may interact more readily with small aquatic organisms and enhance exposure through ingestion, surface contact, and the release of particle-associated chemicals [28,29,30,31]. Suspended APPs may also serve as a continuing source of dissolved contaminants during exposure, thereby creating exposure conditions distinct from those associated with the membrane-filtered leachate. Similar patterns have been reported for antifouling paint particles, hydroblasting residues, and hull-cleaning wastewater, in which particle-containing materials exhibited greater toxicity than their corresponding dissolved fractions [32,33,34]. Thus, direct particle interactions, exposure to particle-associated constituents, residual material passing through the filtration step, and continued contaminant release from suspended particles may all contribute to the difference between the two exposure conditions. These processes are not mutually exclusive and cannot be separated by the present experimental design.

A limitation of the present exposure design is that APP concentrations were expressed as nominal initial loadings rather than analytically measured suspended-particle concentrations. Given the heterogeneous particle-size distribution of the APPs, particularly the presence of relatively large particles, settling and redistribution likely occurred during the static exposure period. Consequently, the suspended APP concentration experienced by the organisms may have varied over time and may have been lower than the nominal concentration. The observed concentration–response relationships should therefore be interpreted in relation to the initial nominal APP loading rather than as responses to a constant suspended-particle concentration. Environmental factors including particle aggregation, salinity, organic matter, suspended solids, hydrodynamics, and resuspension can further influence particle fate and contaminant release. Daily renewal may also have affected particles and dissolved constituents differently, potentially altering exposure composition over time.

The developmental exposure conditions may additionally have been influenced by the presence of *Tetraselmis* sp., which could interact with APPs or dissolved contaminants through adsorption, aggregation, or food-associated uptake. Such interactions may alter the effective exposure experienced by the copepods and could partially attenuate direct interactions between APPs and the organisms. The algae were not pre-exposed to APPs or leachates before addition to the test media. Because subsequent algae–APP–contaminant interactions were not quantified, their magnitude and direction remain uncertain. The same algal concentration was supplied to all treatments, standardizing food provision but not necessarily exposure dynamics.

The environmental relevance of the tested concentrations should likewise be considered in context. Zhang et al. [7] reported 330.25 mg L^−1^ APPs in wastewater from ultra-high-pressure ship-hull derusting, while Kim et al. [35] reported 24.7 mg L^−1^ before and 1.2 mg L^−1^ after treatment of in-water hull-cleaning wastewater. These measurements indicate substantial particle loads in source effluents, but do not represent concentrations in receiving coastal waters. Similarly, the 97.3–249 mg L^−1^ total suspended solids reported in hull-cleaning effluents by Soon et al. [36] provide context for overall particulate loads rather than APP-specific concentrations. In Jinhae Bay, Song et al. [37] reported 94 ± 68 paint-resin particles L^−1^ in the sea-surface microlayer; however, these number-based measurements cannot be directly compared with the mass-based APP concentrations used here.

Accordingly, the present experiments are best viewed as laboratory hazard characterization rather than a simulation of typical ambient coastal exposure. Although some source-effluent concentrations exceed the suspension concentrations tested here, such numerical comparisons do not indicate equivalent biological exposure because particle composition, size distribution, dilution, and exposure duration differ. Likewise, the lower concentrations used in the developmental assay should not be considered environmentally representative simply because they were sublethal. Rather, the available environmental measurements provide context for the potential magnitude of APP release during hull-maintenance activities, whereas the present experiments characterize biological responses to defined laboratory preparations of this material.

Against this exposure context, the developmental assays provided additional evidence of biological effects at sublethal concentrations. The nauplius-to-copepodid (N–C) transition was more sensitive to APP exposure than overall development to adulthood. The N–C developmental period was prolonged in a concentration-dependent manner under both exposure conditions, whereas the nauplius-to-adult (N–A) developmental period was significantly prolonged only at the highest APP suspension concentration. This pattern suggests that APP exposure may affect early postembryonic development before measurable effects on the time required to reach adulthood become apparent. The sensitivity of early copepod development may be associated with the high energetic and physiological demands of rapid molting, tissue differentiation, and somatic growth, which can be readily disrupted by contaminant-induced stress [15,38,39,40]. Developmental delays have likewise been reported in *T. japonicus* exposed to booster biocides such as zinc pyrithione (ZnPT) and copper pyrithione (CuPT), which prolonged naupliar development and delayed maturation [41,42]. Comparable effects observed in other marine invertebrates exposed to antifouling contaminants further support early developmental processes as sensitive biological targets of coating-derived toxicants [43,44,45,46]. However, because the chemical constituents responsible for the observed responses were not resolved in the present study, the developmental effects cannot be assigned to a particular contaminant or exposure pathway.

The transcriptomic results provided a molecular perspective on these organism-level responses, but were interpreted separately from the developmental endpoint. Exposure to the APP suspension yielded approximately four times the number of differentially expressed genes (DEGs) detected following exposure to the corresponding leachate. Specifically, 474 and 116 DEGs were identified in the suspension-versus-control and leachate-versus-control comparisons, respectively. The difference in DEG number is presented as a descriptive feature of the two datasets rather than as a direct measure of relative toxicity, because DEG counts can be influenced by biological variation, sequencing characteristics, and statistical thresholds. GO enrichment analysis indicated that APP exposure was associated with changes in chromatin-related and developmental processes in addition to cellular stress responses. Both exposure groups showed enrichment of biological processes related to nucleosome assembly, chromatin organization, heterochromatin formation, and larval somatic muscle development, suggesting partially shared transcriptional responses involving chromatin regulation and developmental gene programs. At the same time, the two exposure conditions showed differences in their enriched biological-process terms. In the APP suspension group, additional enrichment terms included DNA transposition, asymmetric cell division, brain development, and cold acclimation, whereas the leachate group showed terms including somatic muscle development, specification of segmental identity in the head, nucleus organization, and regulation of cell-fate specification. These differences indicate that the two exposure preparations were associated with partly distinct transcriptional profiles, but they do not by themselves demonstrate treatment-specific mechanisms or differences in overall molecular toxicity. Alterations in nucleosome- and chromatin-associated processes may reflect transcriptional reprogramming in response to cellular stress [47]. Enrichment of developmental terms is also consistent with the potential disruption of biological processes relevant to postembryonic development, although a direct causal link to the observed developmental delays cannot be established from the transcriptomic data alone. The cold-acclimation term may represent a nonspecific stress-response signature rather than a temperature-specific response. Overall, the GO enrichment patterns indicate both shared and distinct biological-process responses between the APP suspension and leachate, providing molecular-level hypotheses for further investigation.

IPA Toxicity Pathway Analysis further identified several significantly enriched pathways following exposure to the APP suspension, including those associated with cytochrome P450-mediated metabolism, NRF2-mediated oxidative stress responses, mitochondrial membrane function, and cell-cycle DNA damage checkpoint regulation. Cytochrome P450 enzymes constitute a major phase I biotransformation system in arthropods and respond to a wide range of organic xenobiotics and metals [19,48,49]. Enrichment of CYP-associated pathways is therefore consistent with altered xenobiotic biotransformation and increased cellular demands for detoxification following APP suspension exposure. Similarly, enrichment of the NRF2-mediated oxidative stress response suggests perturbation of redox homeostasis and the recruitment of antioxidant defense processes, which have been widely reported in marine organisms exposed to Cu, Zn, Cd, and antifouling paint-derived contaminants [50,51,52]. Concurrent enrichment of pathways related to mitochondrial membrane function and DNA damage checkpoint regulation further indicates potential perturbations of energy metabolism and genome maintenance. Such molecular responses may be relevant to the physiological stress associated with APP exposure, although a direct causal relationship with the developmental delays was not established. Importantly, the transcriptomic experiment involved a separate 24-h exposure of adult copepods at the highest sublethal concentrations used in the developmental assays. Therefore, these molecular responses should not be interpreted as direct mechanistic explanations for the developmental effects observed in nauplii. In contrast, the APP leachate induced substantially fewer DEGs and no significantly enriched toxicity-related pathways under the applied analysis criteria. These results indicate differences in the molecular profiles associated with the two exposure conditions, while the underlying chemical or physical basis of these differences remains unresolved.

The combined organismal and molecular findings therefore suggest that retaining only the dissolved/leachate fraction may not fully capture the biological responses associated with APP-containing exposure preparations. Hull-cleaning and maintenance activities, including water-jet blasting, grit blasting, and mechanical scraping, can release substantial quantities of paint particles, with fine fractions potentially remaining suspended or passing through conventional wastewater filtration systems [53,54,55]. Once released into marine environments, APPs may be ingested by benthic and pelagic organisms and may influence contaminant exposure through continued leaching, sedimentation, and subsequent resuspension [9,10]. In the present study, the greater mortality and developmental effects observed with the APP suspension, together with differences in the associated transcriptomic profiles, demonstrate that the biological responses to the particle-containing preparation were not fully reproduced by the corresponding membrane-filtered leachate. However, the study does not distinguish whether this difference resulted from physical particle interactions, particle-associated constituents, residual filter-passing material, continued chemical release, or a combination of these processes. Future studies incorporating quantitative characterization of APP composition and exposure-media chemistry, measurements of suspended particle concentrations during exposure, and experimental designs that independently manipulate particle presence and chemical composition would help resolve the relative contribution of these factors. Overall, the present findings provide comparative evidence that both particle-containing and leachate-associated exposure pathways warrant consideration when evaluating the biological effects of APPs released during hull-maintenance activities.

## 5. Conclusions

The APP suspension produced greater acute mortality and developmental effects and elicited a distinct transcriptomic response compared with the corresponding leachate in *T. japonicus*. However, these differences cannot be attributed solely to the physical presence of particles, as particle-associated chemicals and other exposure-related factors may also contribute. Overall, the findings indicate that evaluating APP toxicity based only on the dissolved/leachate fraction may not fully capture biological responses to particle-containing exposure preparations. Further studies integrating chemical characterization, particle dynamics, and controlled separation of particulate and dissolved effects are needed to better resolve the mechanisms underlying APP toxicity in marine organisms.

## Data Availability

The original contributions presented in this study are included in the article/Supplementary Material. Further inquiries can be directed to the corresponding author.

## Supplementary Materials

The following supporting information can be downloaded at: https://www.mdpi.com/article/10.3390/toxics14100890/s1; Table S1: Physicochemical characteristics of the environmentally collected antifouling paint particles (APPs), including major ingredients and aquatic ecotoxicological information inferred from the Material Safety Data Sheet (MSDS) and Technical Data Sheet (TDS). Table S2: Differentially expressed genes identified following APP suspension and leachate exposure (absolute fold change ≥ 1.5 and adjusted *p* < 0.10; false discovery rate = 10%). Table S3: Enriched Gene Ontology (GO) terms during APP suspension and leachate exposure. Table S4: IPA toxicity pathway analysis of differentially expressed genes (DEGs) following exposure to APP suspension and leachate. Table S5: Probit regression parameters for 24- and 48-h LC_50_ estimates of *T. japonicus* exposed to APP suspension and leachate.

## Abbreviations

The following abbreviations are used in this manuscript:

APPs	Antifouling Paint Particles
DEGs	Differentially Expressed Genes
GO	Gene Ontology
SEM	Scanning Electron Microscopy
FTIR	Fourier-transform Infrared Spectroscopy
ASW	Artificial seawater
COI	Cytochrome c oxidase subunit I
mRNA	Messenger RNA
cDNA	Complementary DNA
FDR	False Discovery Rate
TMM	Trimmed mean of M-values
IPA	Ingenuity Pathway Analysis
N–C	Nauplius-to-copepodid
N–A	Nauplius-to-adult
