# Supplementary material for: Comparative Toxicity of Antifouling Paint Particle Suspensions and Dissolved Leachates in the Marine Copepod Tigriopus japonicus
Source: Toxics. 2026 Oct 6;14(10):890. doi: 10.3390/toxics14100890 (PMC13709851; doi:10.3390/toxics14100890)
Supplement: Supplementary file 1 [file toxics-14-00890-s001.zip › Supplementary data.pdf]

Supplementary data

Comparative Toxicity of Antifouling Paint Particle Suspensions and Dissolved Leachates in the Marine Copepod *Tigriopus japonicus*

Sung-Eun Hong[1,†], Bhavya Kachiprath[1,†], Duck-Hyun Kim[1], Harim Jeong[1], Chae Hwa Kim[2], Tae Hee Kim[2], Chang-Bum Jeong[1,*]

[1]*Department of Marine Science, College of Natural Sciences, Incheon National University, Incheon 22012, Republic of Korea*

[2]*Textile Innovation R&D Department, Korea Institute of Industrial Technology, Ansan 15588, Republic of Korea*

[†]These authors contributed equally to this work.

[*]Corresponding author.

E-mail: cbjeong@inu.ac.kr (C.-B. Jeong)

Supplementary Tables

Table S1. Physicochemical characteristics of the environmentally collected antifouling paint particles (APPs), including major ingradients and aquatic ecotoxicological information inferred from the Material Safety Data Sheet (MSDS) and Technical Data Sheet (TDS).

Table S2. Differentially expressed genes identified following APP suspension and leachate exposure (absolute fold change ≥ 1.5 and adjusted $P < 0.10$; false discovery rate = 10%).

Table S3. Enriched gene ontology (GO) terms during APP suspension and leachate exposure.

Table S4. IPA toxicity pathway analysis of differentially expressed genes (DEGs) following exposure to APP suspension and leachate.

Table S5. Probit regression parameters for 24- and 48-h LC_{50} estimates of *T. japonicus* exposed to APP suspension and leachate.
